# Decoding the Chemical Language of Ribosomally Synthesized and Post‐Translationally Modified Peptides from the Untapped Archaea Domain

**DOI:** 10.1002/anie.202501074

**Published:** 2025-04-14

**Authors:** Zhi‐Man Song, Cunlei Cai, Ying Gao, Xiaoqian Lin, Qian Yang, Dengwei Zhang, Gengfan Wu, Haoyu Liang, Qianlin Zhuo, Junliang Zhang, Peiyan Cai, Haibo Jiang, Wenhua Liu, Yong‐Xin Li

**Affiliations:** ^1^ Department of Chemistry and The Swire Institute of Marine Science The University of Hong Kong Pokfulam Road Hong Kong China; ^2^ Chemistry and Chemical Engineering Guangdong Laboratory Shantou 515031 China

**Keywords:** Archaeal secondary metabolites, Chemical biology, Lanthipeptide, RiPP

## Abstract

Chemical communication is crucial in ecosystems with complex microbial communities. However, the difficulties inherent to the cultivation of archaea have led to a limited understanding of their chemical language, especially regarding the structure diversity and function of secondary metabolites (SMs). Our in‐depth exploration into the biosynthetic potential of archaea has unveiled the previously unexplored biosynthetic capabilities and chemical diversity of archaeal ribosomally synthesized and post‐translationally modified peptides (RiPPs). Through the first application of heterologous expression in archaeal SM discovery, we have identified 24 lanthipeptides, including a distinctive type featuring diamino‐dicarboxylic termini. It highlights the uniqueness of archaeal biosynthetic pathways and significantly expands the chemical landscape of archaeal SMs. Additionally, archaeal lanthipeptides demonstrate antagonistic activity against haloarchaea, mediating the unique biotic interaction in the halophilic niche. They showcase a new ecological role of RiPPs in enhancing the host's motility by inducing the rod‐shaped cell morphology and upregulating the archaellin gene expression, facilitating the archaeal interaction with abiotic environments. These discoveries broaden our understanding of archaeal chemical language and provide promising prospects for future exploration of SM‐mediated interaction.

## Graphical abstract

Secondary metabolites (SMs) are essential across all life domains, yet those originating from the Archaea domain remain poorly understood. Here, the systematic genome mining and the pioneering heterologous expression of archaeal SMs have revealed the chemical landscape of archaeal lanthipeptides, showing both canonical and non‐canonical forms. These lanthipeptides exhibit antagonistic activity and activate the host's motility, likely shaping the archaeal community and mediating environmental adaptation.

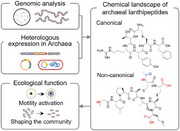

## Introduction

Archaea, the third domain of life, have evolved distinctly from bacteria and eukaryotes.^[^ ^1^ ^]^ Their genetic makeup combines features of both domains, such as bacterial operons and eukaryotic histone‐like proteins,^[^ ^2^ ^]^ alongside unique adaptations like ether‐linked membrane lipids and specialized metabolic pathways that enable survival in extreme environments.^[^ ^3^, ^4^, ^5^, ^6^, ^7^, ^8^, ^9^, ^10^ ^]^ These genomic and biochemical traits, combined with their ubiquity in diverse ecosystems, highlight the unique and essential roles of Archaea.^[^ ^11^ ^]^ However, their cultivation in laboratory settings remains challenging due to specialized growth requirements.^[^ ^11^, ^12^, ^13^ ^]^ They have developed various mechanisms to thrive in different environments, involving direct and indirect interactions with biotic and abiotic factors.^[^ ^14^, ^15^, ^16^, ^17^, ^18^, ^19^, ^20^ ^]^ To date, our understanding of chemical‐mediated interactions in archaea lags far behind that of bacteria and fungi, with knowledge primarily limited to certain UV‐protective carotenoids,^[^ ^21^ ^]^ putative siderophores,^[^ ^22^ ^]^ and quorum‐sensing molecules.^[^ ^23^, ^24^ ^]^

Archaea possess a variety of enzymes and metabolic pathways distinct from those in bacteria,^[^ ^3^, ^4^, ^5^, ^6^, ^7^, ^8^, ^9^, ^10^ ^]^ marking them as a promising and largely untapped reservoir of novel enzymes and secondary metabolites (SMs). However, due to the challenges in archaeal cultivation and genetic manipulation, the chemical landscape, biosynthesis, and ecological function of archaeal metabolites remain largely unexplored.^[^ ^11^, ^13^, ^25^ ^]^ Recent bioinformatic analyses of archaeal SMs have revealed the presence of ribosomally synthesized and post‐translationally modified peptide (RiPP) biosynthetic gene clusters (BGCs), including YcaO‐related RiPP,^[^ ^26^ ^]^ radical S‐adenosylmethionine (rSAM)‐dependent enzyme‐modified RiPP,^[^ ^27^, ^28^ ^]^ and lanthipeptide^[^ ^29^, ^30^, ^31^ ^]^ biosynthetic pathways. Despite thousands of predicted BGCs and numerous attempts of heterologous expression and wild‐type strain fermentation, functional characterization of these putative BGCs remains limited, with only two lanthipeptides from Haloarchaea experimentally validated in previous research.^[^ ^31^ ^]^ Expressing archaeal biosynthetic pathways heterologously in bacterial hosts can be problematic due to distinct metabolic features, potentially leading to improper folding, low expression levels, or inactive archaeal enzymes.^[^ ^32^ ^]^ The scarcity of SMs from even well‐defined archaeal RiPP BGCs,^[^ ^26^, ^27^, ^28^, ^29^, ^31^ ^]^ alongside the challenges involved in their heterologous expression in model bacterial hosts, underscores the necessity for new strategies in discovering archaeal metabolites.

Uncovering the yet unexplored chemistry of archaeal SMs will provide crucial insights into their chemically mediated interactions, invigorating the relatively underexplored field of archaeal chemical biology. Here, our study represents the systematic investigation and the first heterologous expression‐based discovery of RiPPs in archaea, aiming to uncover their biosynthetic potential, unique chemical landscape, and ecological functions. We successfully identified 24 classic and noncanonical lanthipeptides of RiPPs which originated from eleven BGCs by combining the haloarchaeal heterologous expression system with biosynthetic rule‐guided metabolomic analysis.^[^ ^33^ ^]^ More importantly, our research unveiled a new ecological function of lanthipeptides in stimulating the host's motility by promoting a rod‐like morphology and upregulating archaellin gene expression. Our study represents a substantial advancement in understanding the significant ecological contributions of SMs within the archaeal community and paves the way for future research in archaeal chemical biology and chemical ecology.

## Results

### Genome Mining Reveals the Largely Untapped Biosynthetic Potential of Archaeal RiPPs

**Figure 1:**
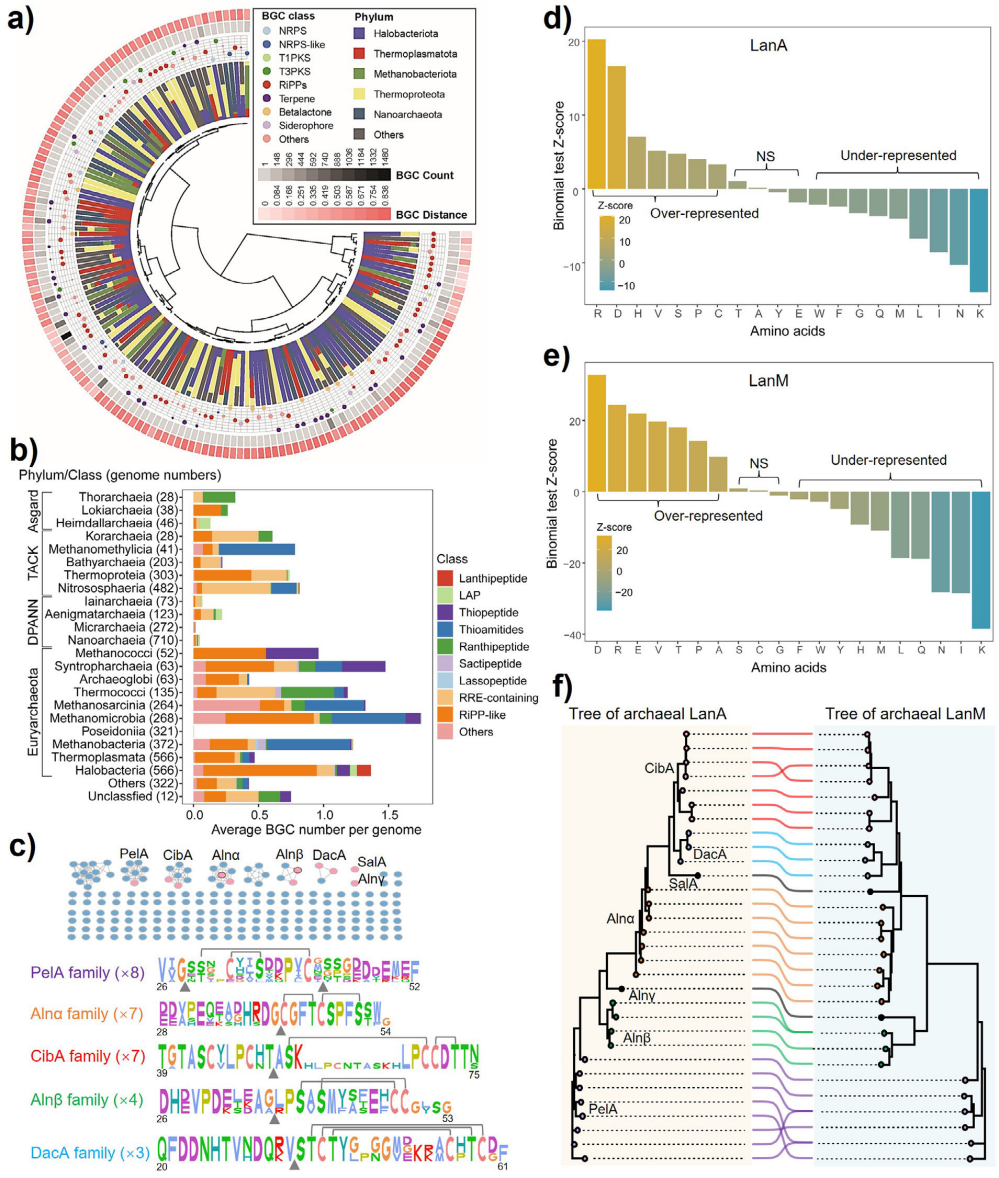
Uncovering the biosynthetic potential of archaeal RiPPs. a) The novelty, diversity, and distribution of archaeal BGCs. Layers from innermost to outermost show hierarchical clustering of the 148 gene cluster clans (GCCs) based on the BGC distances, phylum distribution for each clan, the proportion of different BGC classes for each clan, BGC count, and average cosine distance to known BGCs. b) The distribution of RiPP BGCs in the domain of Archaea at the class level. c) The SSN analysis of putative archaeal LanAs and sequence logos of experimentally verified LanA families. Sequences with 100% identity are grouped into a single node. Pink nodes represent the identification of heterogeneous expression products or wild‐type natural products in this study. Black circles represent previously verified lanthipeptides. Lines show the validated crosslinks between C and S/T residues. The main cleavage sites observed between the leader and core peptides are displayed as triangles below the LanAs. d,e) Binomial tests were conducted to compare the proportions of all 20 amino acids in d) LanAs and e) LanMs between archaeal and bacterial counterparts. *Z*‐scores were calculated relative to archaeal LanAs and LanMs, where *Z* > 2.0 indicated significant enrichment of a given amino acid in Archaea (“Over‐represented”), *Z* < ‐2.0 indicated significant depletion (“Under‐represented”), and some amino acids showed no significant biases (“NS”). f) Co‐phylogenomic analysis of archaeal LanAs (left) and LanMs (right) from verified SSN families. The nodes and lines are colored according to their LanA families in c). Two black notes represent verified orphan notes in the SSN analysis, SalA and Alnγ.

Our previous genomic analysis indicated that archaea contain a relatively small number of BGCs per genome compared to the extensively studied and well‐documented bacterial counterparts.^[^ ^31^ ^]^ They encode a broad range of SM classes, such as RiPPs, terpenes, nonribosomal peptides (NRPs), and polyketides (PKs). Our initial fermentation‐based discovery identified a new lanthipeptide from Haloarchaea, marking the first discovery of lantibiotic in the archaeal domain.^[^ ^31^ ^]^ In the current study, we further applied the antiSMASH 7.0 tool^[^ ^34^ ^]^ to examine 11644 archaeal genomes from the NCBI database to delve deeper into the biosynthetic landscape, diversity, and novelty of archaeal RiPPs. From 3451 representative archaeal genomes, 7731 BGCs were identified, mainly from phyla Halobacteriota (3379, 43.7%), Thermoplasmatota (1359, 17.6%), Methanobacteriota (1271, 16.4%), Thermoproteota (962, 12.4%), and Nanoarchaeota (221, 2.9%) (Figure 1a and Supplementary Data 1). Among them, RiPPs (3599, 46.6%), Terpenes (1990, 25.7%), and NRPs (581, 7.5%) emerged as dominant BGCs in the domain of Archaea (Figure 1a). These BGCs exhibited significant novelty compared to known BGCs from MIBiG,^[^ ^35^ ^]^ indicating a largely unexplored source for natural product discovery.

Phylogenetic distribution analysis unveiled a remarkable diversity within RiPP BGCs, widely distributed across the Asgard, TACK, and DPANN superphyla and predominantly within Euryarchaeota (Figure 1b). Among these abundant RiPP classes are YcaO‐related RiPPs (thioamitides and thiopeptides), rSAM‐dependent enzyme‐modified RiPPs (ranthipeptides and sactipeptides), lanthipeptides, and lassopeptides (Figure 1b). Although RiPP BGCs are widely distributed throughout different phyla/classes, the scarcity of identified archaeal RiPP products hinders us from elucidating their unique chemical structure and putative ecological functions. Unlike other archaeal RiPP BGCs, class II lanthipeptide BGCs, defined by the presence of the lanthipeptide synthase LanM, are exclusively distributed in Haloarchaea (refer to class Halobacteria) within our dataset, hinting at a niche‐specific association (Figure 1b, Supplementary Data 1 and 2). Consequently, we constructed an updated library in silico to decipher the chemical landscape of archaeal lanthipeptides. This library contains 353 putative precursors (LanAs) and 110 lanthipeptide synthase LanMs from 102 archaeal lanthipeptide BGCs (Supplementary Data 2). Among these, 67 deduplicated archaeal LanMs, exclusively present in Haloarchaea, are mainly from the genera *Halorussus* (26, 38.8%) and *Haloferax* (14, 20.9%), followed by *Haladaptatus* (5, 7.5%), *Halobacteriales* (5, 7.5%), and *Natrinema* (5, 7.5%) (Figure S1a and Supplementary Data 2). Moreover, 196 unique LanA sequences have been categorized into 157 families through sequence similarity network (SSN) analysis (Figure 1c), highlighting untapped chemical space for lanthipeptide discovery.

### Co‐Evolved Archaeal LanMs and LanAs Exhibit Environmental Adaptation

A collective analysis of the phylogenetic tree, amino acid composition bias, SSN, and sequence logo indicates distinct chemical signatures of archaeal lanthipeptides compared to their bacterial counterparts (Figure 1 and Figures S1 and S2). The phylogenetic tree of archaeal LanMs highlighted their evolutionary divergence from bacterial LanMs (Figure S1b and Supplementary Data 2). The tree of archaeal LanAs exhibited two primary clades: one encompassing our previously characterized LanAs, Alnα and Alnβ, and another unexplored clade that offers prospects for further investigation (Figure S2). Additionally, archaeal LanAs are predicted to be shorter,^[^ ^31^ ^]^ with a medium length of 51 amino acids (aa) compared to 58 aa for bacterial LanAs (Mann–Whitney U test, *P* < 0.001, Supplementary Data 2). Furthermore, archaeal LanAs comprise a higher portion of C and S instead of T residues than bacterial LanAs (Figure 1c,d), which are crucial conserved residues for thioether ring formation in lanthipeptide biosynthesis. More importantly, the unique amino acid composition bias observed in archaeal LanAs and LanMs, as opposed to their bacterial counterparts, supports the hypothesis that they underwent eco‐evolution in response to hypersaline environments (Figure 1d,e). Specifically, archaeal LanMs and LanAs evolved to over‐represent D, V, R, and P residues while under‐representing K, I, N, Q, M, L, W, and F residues (Figure 1d,e). The increased abundance of D/E residues and the reduced portion of K residue is linked to the “salt‐in” strategy of Haloarchaea, enabling interactions with cations in their high‐salinity environment.^[^ ^17^, ^36^ ^]^ Furthermore, the reduced occurrence of hydrophobic amino acids (I, L, M, W) may increase random‐coil structure formation, thereby preventing protein aggregation or inactivation in low‐water environments. The unusually high prevalence of basic R residues in archaeal LanMs and LanAs may represent a distinct evolutionary signature and potential functional determinant of archaeal lanthipeptides. To our knowledge, this distinctive feature has not been observed in any other reported halophilic enzyme systems or proteomic studies to date.^[^ ^36^ ^]^ These results imply that archaeal LanAs, co‐evolving with LanMs as a part of environmental adaptation (Figure 1f), may enhance the chemical diversity of lanthipeptides exclusive to Haloarchaea, thereby playing significant ecological roles in halophilic environments.

### Heterologous Expression‐based Discovery of Canonical Archaeal Lanthipeptides

**Figure 2:**
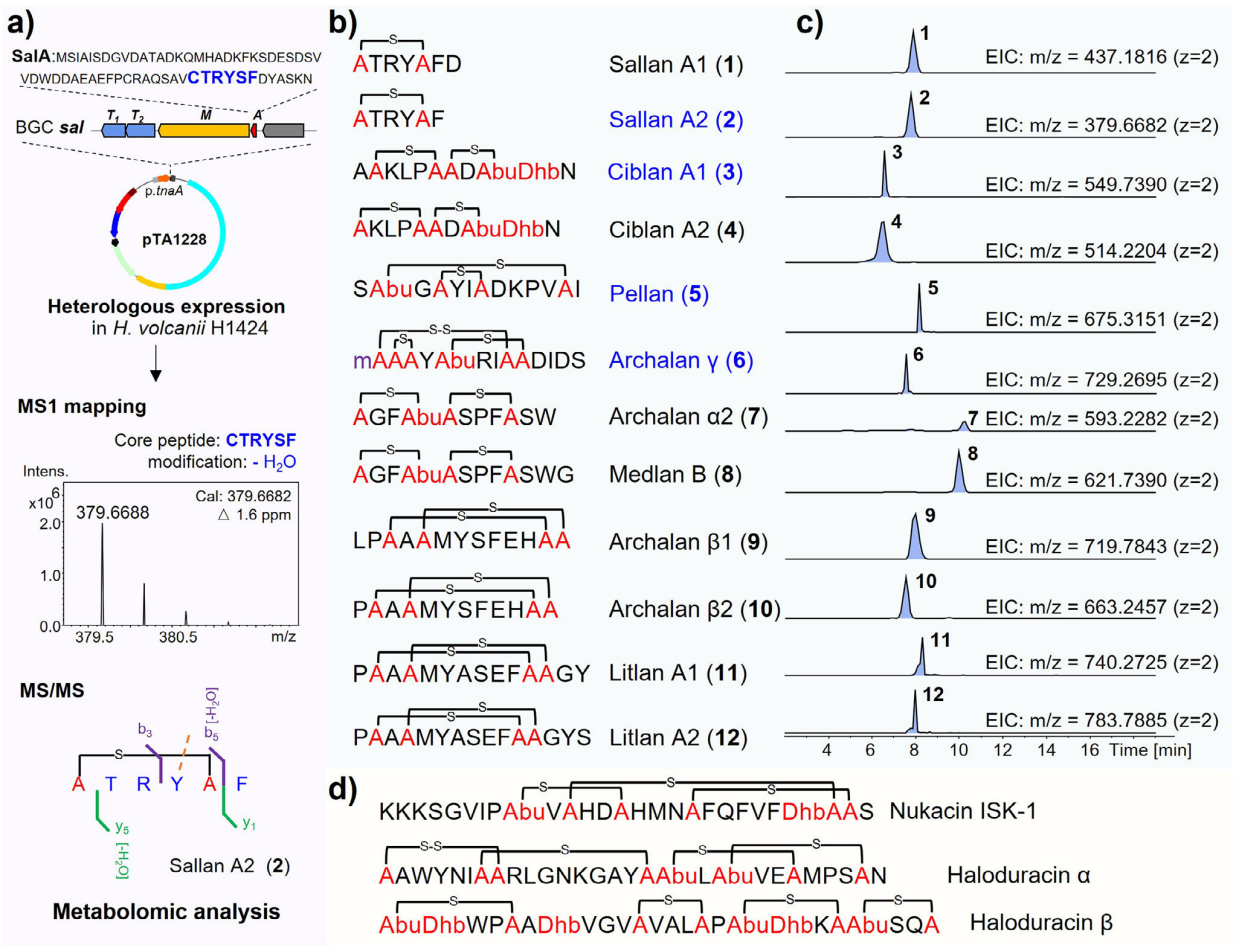
Identifying archaeal class II lanthipeptides through heterologous expression and biosynthetic rule‐guided metabolomic analysis. a) The workflow for heterologous expression‐based discovery of archaeal lanthipeptide in *H. volcanii* H1424, in conjunction with metabolomic analysis, is illustrated by the example of sallan A2 (**2**). Classical lanthipeptides b,c) with different modifications (“m” in purple represents methylation) were identified via HRMS. Structures confirmed through NMR analysis or further predicted by chemical derivatization are marked in blue. d) Representative bacterial class II lanthipeptides.

Moving beyond the traditional method of natural product discovery through wild‐type cultivation, our initial attempts to heterologously express archaeal lanthipeptide BGCs in the model bacterial hosts (*Escherichia coli* and *Bacillus subtilis*) were unsuccessful. This result was associated with our evolutionary and amino acid bias analysis, suggesting a possible unique biosynthetic characteristic in archaeal lanthipeptides. To solve this problem, we utilized heterologous expression in haloarchaea, *Haloferax volcanii* H1424, using the pTA1228 vector (Figure 2a, Table S2 and Supplementary Data 3).^[^ ^37^, ^38^ ^]^ The efficacy of this procedure was confirmed by the successful expression of our previously validated lanthipeptide BGCs,^[^ ^31^ ^]^ *alnα* and *alnβ*, which resulted in the production of corresponding analogs of the natural lanthipeptides, archalan α2 (**7**), β1 (**9**), and β2 (**10)** (Figure 2b,c and Figures S3 and S4). It marks the first successful validation of archaeal BGCs via heterologous expression (Figure 2), laying the groundwork for subsequent expression of additional archaeal lanthipeptide BGCs.

Based on this approach, we selected eleven additional BGCs for investigation and detected putative lanthipeptide signals in the crude extracts of nine of the corresponding recombinant strains. Among these, seven BGCs were successfully linked with their canonical lanthipeptide products, including sallans from *sal* BGC, ciblans from *cib1* and *cib2* BGCs, pellan from *pel* BGC, archalan γ from *alnγ* BGC, medlan B from *medb* BGC, and litlans from *lit* BGC (Figures 1c and 2, and Figures S5–S36). It is worth mentioning that sallans, pellan, and litlans were absent in their corresponding wild‐type strain fermentation extracts, highlighting the effectiveness of the archaeal heterologous expression system in unearthing cryptical archaeal metabolites.

**Figure 3:**
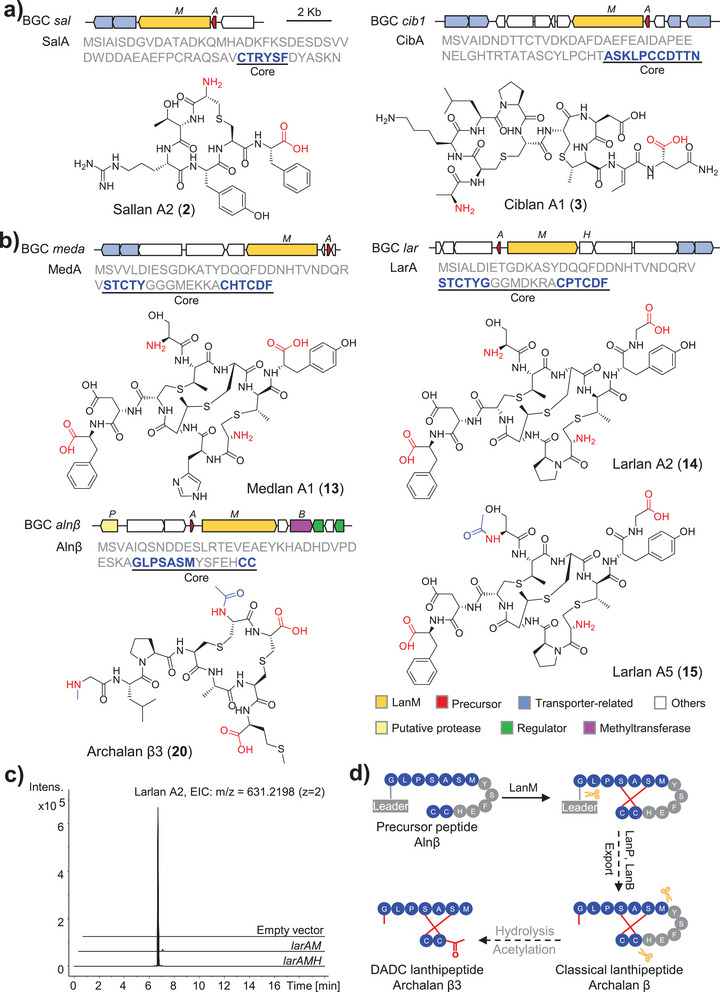
Novel chemistry and biosynthesis of archaeal lanthipeptides. a,b) The BGC constructions, precursors, and chemical structures of classical and DADC lanthipeptides. The excised residues within precursor peptides are depicted in gray, while the enduring core peptide residues are illustrated in blue. The C/N termini in the structure are in red, and specific modifications at the N‐terminus are in blue. The stereo structures of medlan A1 (**13**) and Larlan A5 (**15**) are deduced based on the stereo structure of Larlan A2 (**14**). c) LC‐MS analysis evaluated the production of larlan A2 in the in vivo reconstitution of BGC *lar* with/without gene *larH*. d) Proposed biosynthetic pathway of DADC lanthipeptides, demonstrated by archalan β3.

To pinpoint low‐yield BGC‐encoded peptides within the metabolomic data, we mapped the calculated mass data, which was predicted based on lanthipeptide post‐translational modification rules,^[^ ^33^ ^]^ with the high‐resolution mass spectrometry (HRMS) data to identify corresponding hits (Figure 2a). For instance, HRMS analysis of the crude extracts from the recombinant strain harboring the BGC *sal* (*Halorussus salinus* YJ‐37‐H) exposed low‐molecular‐weight peptidic signals (Figure S5). The MS1 matching process revealed that a hit of [M + 2H]^2+^  =  379.6688 (**2**) matched the calculated mass data well of “CTRYSF” with one dehydration (calculated [M + 2H]^2+^ = 379.6682), linking this low‐yield peptide to precursor SalA (Figure 2a and Figure S5). Furthermore, the MS/MS data confirmed a C‐S crosslink between Cys1 and Ser5 (b_5_, ‐H_2_O, ‐18 Da) in **2** (Figure S5). Additionally, sallan A1 (**1**, observed [M + 2H]^2+^ = 437.1816, calculated [M + 2H]^2+^ = 437.1816, Δ  = 0.0 ppm) was predicted to be an analog of **2**, differing by an extra D residue at the N‐terminus (y_2_, “FD” residue, Figure S5). More importantly, 2D nuclear magnetic resonance (NMR) data of purified **2** confirmed the thioether ring in Cys1‐Ser5 (*δ*_H_ = 2.96, 3.31 ppm to *δ*_C_ = 35.9 ppm and *δ*_H_ = 3.07, 3.24 ppm to *δ*_C_ = 34.3 ppm) (Figure 3, Figure S6 and Table S3), which was consistent with the result of metabolomic analysis. The stereochemical analysis confirmed that all unmodified amino acids of **2** were of L configuration and the Lan residue was of DL (2*S*,6*R*) configuration (Figure 3a, Figure S7, and Table S4). Notably, **2** with only six amino acids represents the smallest natural lanthipeptide to date.

Ciblan A1 (**3**, BGC *cib1*, *Haladaptatus cibarius* JCM 19505) and ciblan A2 (**4**, BGC *cib2*, *H. denitrificans* DSM 4425) were predicted with a similar modification on their corresponding core peptides (Figure S8). The modification includes bicyclic ring structure and one unsaturated amino acid Dhb residue (Figure 2), evidenced by a series of b ions (b_6_/b_5_ for the first ring and b_9_/b_8_ for the second ring in ciblan A1/A2) and z_2_ fragment in MS/MS analysis (Figure S8). The NMR data of purified **3** exhibited the presence of Dhb residue (C_α_ = 131.0 ppm, C_β_ = 130.9 ppm, H_β_ = 6.66 ppm, C_γ_ = 13.4 ppm, H_γ_ = 1.86 ppm), along with two thioether rings in Ser2‐Cys6 (*δ*_H_ = 2.97, 2.92 ppm of Ala6 to *δ*_C_ = 35.0 ppm of Ala2) and Cys7‐Thr9 (*δ*_H_ = 2.98 ppm of Abu9 to *δ*_C_ = 41.2 ppm of Ala7) (Figure 3a, Figure S9 and Table S3). The NMR‐verified structure further supported the deductions from the MS/MS analysis (Figure S8). The stereochemical analysis confirmed that all unmodified amino acids of **3** were of L configuration and the thioether rings were of DL‐Lan (2*S*,6*R*) and DL‐MeLan (2*S*,3*S*,6*R*) configurations (Figure 3a, Figure S7, and Table S4).

The full desulfurization and MS/MS analysis of pellan (**5**, BGC *pel, H. pelagicus* RC‐68) deduced the presence of two dehydrations within residues Thr2, Cys4, Ser7, and Cys12, and proved intertwined ring structure in the core peptide of PelA (STGCYISDKPVCI, Figure S10). The partial desulfurization and dithiothreitol reduction products of archalan γ (**6**, BGC *alnγ*, *H. salinus* YJ‐37‐H) supported one methylation on Cys1, a disulfide bond between Cys1 and Cys8 and C‐S crosslinking in Ser2‐Cys3 and Thr5‐Cys9 (CSCYTRICCDIDS, Figures S11–S17). This structure expands the limited number of lanthipeptides possessing the smallest thioether ring formed by the adjacent Cys and Ser/Thr residues.^[^ ^39^ ^]^ Archalan α2 (**7**, BGC *alnα*, *H. salinus* YJ‐37‐H) and medlan B (**8**, BGC *medb*, *H. mediterranei* ATCC 33500) contained two thioether rings, supported by their key MS fragments of b_4_ and b_9_ ions (Figure S3), in line with the previously reported structure of archalan α.^[^ ^31^ ^]^ Archalan β1–2 (**9** and **10**, BGC *alnβ*, *H. salinus* YJ‐37‐H) and litlan A1‐2 (**11** and **12,** BGC *lit*, *H. litoreus* JCM31109) were deduced to possess thioether crosslinking patterns similar to the previously reported archalan β.^[^ ^31^ ^]^ Their putative structures feature intertwined thioether rings, with MS/MS fragments originating from the N/C‐terminal residues outside the rings (Figures S4 and S18).

The identified structures of archaeal lanthipeptides emphasize the advantages and feasibility of utilizing heterologous expression in conjunction with biosynthetic rule‐guided metabolomic analysis to unveil the latent chemistry in Archaea, particularly those with limited yields. Furthermore, the chemical compositions of mature archaeal lanthipeptides demonstrate that they are typically shorter than bacterial lanthipeptides, such as nukacin ISK‐1 and haloduracins (Figure 2d). Despite being short in length, their bicyclic and intertwined crosslinking patterns and modifications (e.g., disulfide bond, methylation, dehydration, hydroxylation) are as intricate as those found in bacterial lanthipeptides.

### Characterization of Unique Archaeal Lanthipeptides Featuring Unprecedented Maturation

Apart from the classical lanthipeptides, we detected non‐canonical peptide signals originating from the *meda* and *lar* BGCs (Figure 3 and Figures S19–S29). These signals were identified in the extracts of both wild‐type and recombinant strains, confirming their biosynthetic connection to these BGCs. Unlike classical lanthipeptides, which can be precisely characterized through MS/MS analysis to predict core peptide sequences and modifications, these peptide signals provided only short amino acid fragments lacking consistent patterns. For example, an “FD” fragment from the C‐terminus of LarA (CPTCDF), a “Y” residue also from C‐terminus, and an “S” residue from N‐terminus (STCTY) were identified (Figure 3b and Figure S24).

To fully characterize their structures, 3.2 mg of **13** (observed [M + 3H]^3+^ = 415.4769, calculated [M + 3H]^3+^ = 415.4772, Δ  = 0.7 ppm) from the wild‐type strain, 3.5 mg of **14** (observed [M + 2H]^2+^ = 631.2194, calculated [M + 2H]^2+^ = 631.2198, Δ  = 0.6 ppm), and 2.0 mg of **15** (observed [M + 2H]^2+^ = 652.2301, calculated [M + 2H]^2+^ = 652.2251, Δ  = 7.7 ppm) from the recombinant strain were purified, which were named medlan A1 (**13**), larlan A2 (**14**), and larlan A5 (**15**), respectively. Further NMR spectroscopy analysis revealed that these lanthipeptides comprised two antiparallel peptide chains crosslinked by thioether bonds, thereby defining them as a novel lanthipeptide subfamily (Figure 3b, Figures S20–S22 and Table S3). These crosslinked patterns expose diamino‐dicarboxylic termini with certain amino acids within the thioether rings being partially cleaved, leading to the name of DADC lanthipeptides (Figure 3b). In addition, medlan A2 (**16**), larlans A1 (**17**), A3 (**18**), and A4 (**19**) are analogs of medlan A1 (**13**) or larlan A2 (**14**) and deduced to exhibit variable N/C‐terminal residues extending outside the thioether rings, possibly due to hydrolysis by unknown aminopeptidases (Figures S23–S29). The three MeLan residues in medlans and larlans increased the complexity of the stereochemical analysis, necessitating the construction of single mutations of Thr to Ser in precursor LarA. To assign the absolute configuration of each MeLan residue in larlan A2, 0.3 mg of each mutant (T2S and T4S) were purified for advanced Marfey's analysis and compared the results with four MeLan standards (Figure S7 and Table S4).^[^ ^40^, ^41^ ^]^ Stereochemical analysis confirmed that the thioether rings formed between Thr2 and Cys10, Thr9 and Cys3 within larlan A2 were of LL‐MeLan (2*R*,3*R*,6*R*) configuration, whereas the ring between Thr4 and Cys7 was of DL‐MeLan (2*S*,3*S*,6*R*) configuration (Figure 3b and Figure S7).

To find out whether other lanthipeptides also employ a similar maturation strategy, the MS/MS data of both wild‐type and recombinant strains were meticulously analyzed. The metabolomic profile of *H. salinus* YJ‐37‐H, harboring six lanthipeptide BGCs, displayed mass signals with a fragmental pattern resembling larlans and medlans (Figures S30–S36). A representative compound of **20** (observed [M + 2H]^2+^ = 453.6800, calculated [M + 2H]^2+^ = 453.6796, Δ  = 0.9 ppm) was obtained for NMR (1.0 mg) and advanced Marfey's analysis (Figures S31 and S32). As anticipated, archalan β3 (**20**), derived from BGC *alnβ*, shares the same characteristics as DADC lanthipeptides. The putative structures of other derivatives, archalans β4–7 (**21**–**24**), were also detected and predicted by LC‐MS/MS with additional modifications or N/C‐terminal stretches of residues outside the rings (Figures S33–S36). The advanced Marfey's analysis revealed that the Lan residues of archalan β3 were of LL (2*R*,6*R*) configurations, and all unmodified amino acids were of L configurations, except for the achiral Gly residue (Figure 3b, Figure S7, and Table S4).

Identifying this particular lanthipeptide subfamily in Archaea highlights the existence of unique biosynthetic pathways and enzymes that are distinct from bacterial counterparts. To characterize the minimal essential genes for efficient biosynthesis of larlan A2, the *lar* BGC was selected for in vivo reconstitution. Co‐expression of *larA* and *larM* produced trace amounts of larlan A2 (Figure 3c), suggesting the potential contributions from generic peptidases in the host. Furthermore, co‐expressing them with the unannotated gene *larH* significantly improved the yield (Figure 3c and Supplementary Data 3), indicating that LarH may act as a regulator, cofactor, chaperone, or possess other unknown function in larlan A2 biosynthesis. In another case, although classical and DADC lanthipeptides were detected in the wild‐type strain, the recombinant strain only yielded classical lanthipeptides of archalan β1–2, despite the incorporation of the putative peptidase from *alnβ* BGC (Figure 3 and Figure S4). This outcome suggests the involvement of extraneous proteases encoded outside the BGC within the genome of the wild‐type strain, pointing toward a complex biosynthetic pathway for DADC lanthipeptides. It likely involves additional hydrolysis steps compared to classical lanthipeptides, possibly facilitated by unidentified proteases that cleave amino acids within the thioether rings (Figure 3d). The exclusive identification of this lanthipeptide subfamily within Archaea showcases the uniqueness of archaeal biosynthetic pathways and enzymatic machinery.

### Archaeal Lanthipeptides Exhibit Anti‐Archaeal and Motility Regulation Activities

Given the chemical diversity and novelty of archaeal lanthipeptides, we next wanted to investigate their ecological functions. Seven lanthipeptide crude extracts obtained from heterologous expression and seven purified lanthipeptides were collected for antimicrobial activity screening against nine haloarchaeal and six bacterial strains (Figure S37a–c). The same gradient fractions from the host containing an empty vector were tested for comparison to exclude the potential influence of halocin produced by the heterologous host.^[^ ^42^ ^]^ Specifically, crude extracts from the heterologous expression of BGCs *medb, cib2*, and *alnα‐γ* exhibited significant anti‐haloarchaeal activity at 100 µg mL^−1^ (Figure S37a). The lanthipeptides from BGCs *alnα* and *medb*, archalan α (**25**), archalan α2 (**7**), and medlan B (**8**), were analogs exhibiting a similar anti‐haloarchaeal spectrum (Figure S37a,b). The purified archalan α (**25**) demonstrated strong inhibition against *H. larsenii* JCM 13917, *H. cibarius* DSM 19505, *H. amylolyticus* JCM 18367, and *Halomicroarcula salina* JCM 18369 at 100 µg mL^−1^ (Figure S37b). Notably, **25** exhibited a minimum inhibitory concentration of 6.25 µg mL^−1^ (5 µM) against *H. salina* JCM 18369 (Figure S37c). The anti‐haloarchaeal activity of archaeal lanthipeptides may be attributed to the characteristics of their habitats, which predominantly host Haloarchaea.^[^ ^43^ ^]^

**Figure 4:**
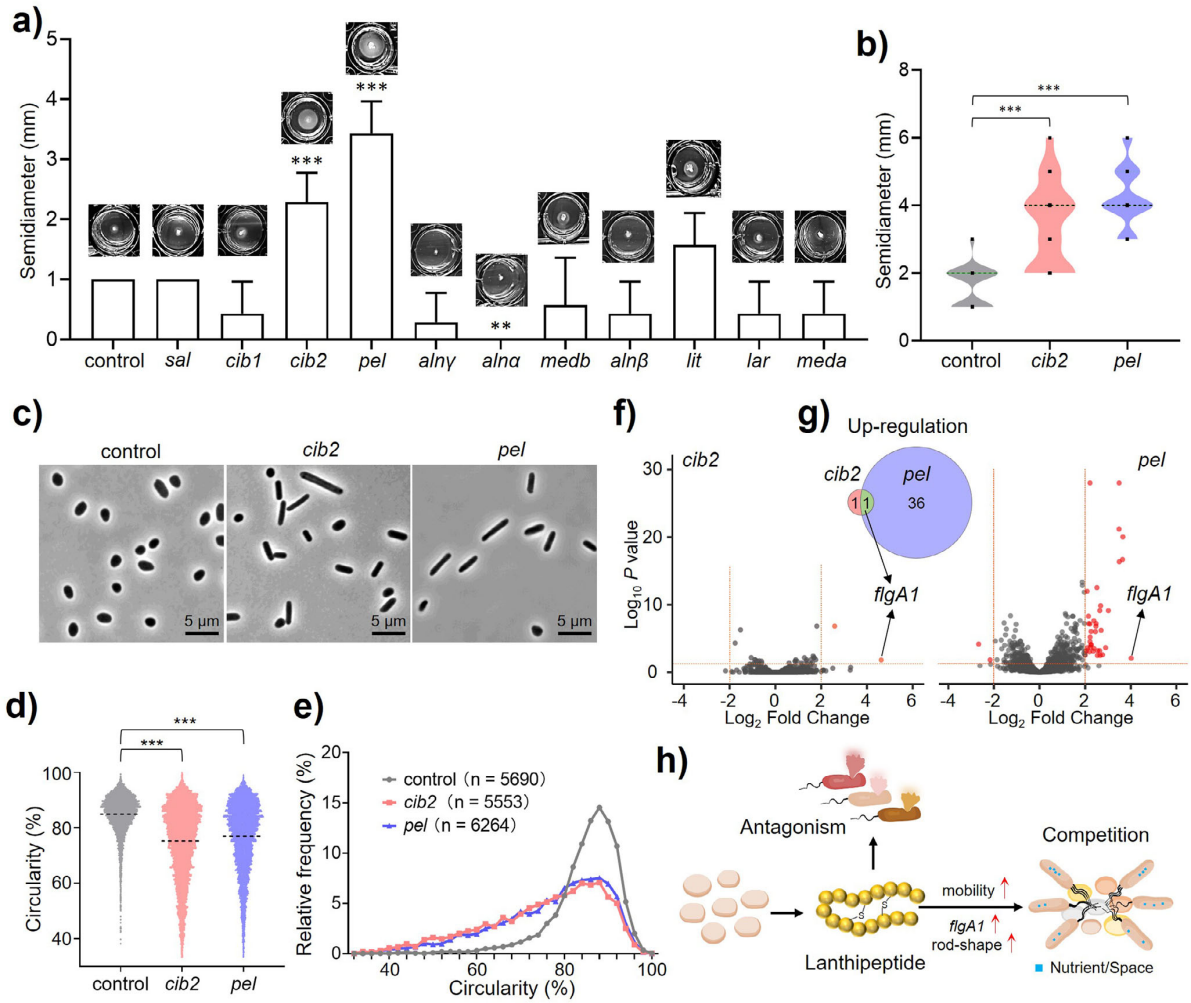
In vivo function of archaeal lanthipeptides. a) The motility assay screening of *H. volcanii* H1424 with empty vector (control) or different lanthipeptide BGCs in 24 well plates with a diameter of 1.56 cm/well. *** *P* < 0.001, ** *P* < 0.05. Each group has three biological replicates. Bars show the mean ± standard deviation (SD), n = 7. b) The violin plot of the motility assay of *H. volcanii* H1424 with empty vector, *cib2* and *pel* BGC, respectively. The median and quartile values are shown as horizontal lines in black and green respectively. Black dots are the aligned experiment data, *** *P* < 0.001, n = 21. Each group has six biological repeats. c) Phase‐contrast images of *H. volcanii* H1424 with empty vector, *cib2*, and *pel* BGC, respectively. d) Cell circularity and e) frequency distribution were calculated from the phase‐contrast microscopy of recombinant strains. Each group has three biological replicates. Lines display the data average of each group, *** *P* < 0.001. f) Volcano plots illustrate the differential expression genes (DEGs) in strains with BGC *cib2* (left) or *pel* (right) compared to the strain with an empty vector. Each group has three biological replicates. g) Venn diagrams depicting the overlap DEGs detected in both strains. h) A proposed mode for the ecological function of archaeal lanthipeptides in antagonistic interaction and nutrient/space competition.

To study the in vivo function of archaeal lanthipeptides, stab‐inoculation screens were conducted to evaluate their impact on host motility. Surprisingly, the motility of three recombinant strains was either positively or negatively affected (Figure 4a). Strains containing BGCs *cib2* and *pel*, responsible for the production of ciblan A2 (**4**) and pellan (**5**), exhibited a significantly larger motility zone than the control (Figure 4b, Figure S37 and Supplementary Data 4). To address whether the motility‐regulatory function of lanthipeptides depends on the growth regulation of the recombinant strain, we evaluated this relationship through time‐course experiments and growth kinetics analysis. The results indicated that *cib2* and *pel* BGCs significantly activated the host's motility without stimulating growth (Figure S37). Conversely, BGC *alnα* suppressed motility through the antagonistic activity of archalan α (**25**) and α2 (**7**), which could impede the host's growth (Figure S37). These findings point to a new ecological role of lanthipeptides, which may reduce the host's reaction time in response to stimuli by regulating motility activity.^[^ ^44^, ^45^, ^46^ ^]^

A comprehensive investigation combining morphological and transcriptomic analysis was conducted to elucidate the mechanisms by which archaeal lanthipeptides enhance motility. Microscopic analysis of recombinant strains demonstrated that lanthipeptides could elevate the frequency of rod‐shaped cell morphology, a characteristic associated with hypermotility (Figure 4c–e and Supplementary Data 5).^[^ ^44^, ^45^, ^46^ ^]^ Additionally, the transcriptomic analysis revealed that the archaellum gene *flgA1*, a major gene responsible for the motility of *H. volcanii*,^[^ ^47^ ^]^ was upregulated in strains possessing BGCs *cib2* or *pel* (Figure 4f,g and Supplementary Data 6). These findings support the hypothesis that lanthipeptides may promote motility by inducing rod‐shaped cell morphology and upregulating the expression of archaellin (Figure 4h).

## Discussion

Archaea, which predominantly survive in extreme environments, are reported to have unique metabolic pathways with eco‐evolutionary strategies distinct from bacteria and fungi.^[^ ^11^, ^17^ ^]^ A notable portion of archaeal BGCs consists of RiPPs and terpenes,^[^ ^31^ ^]^ as identified in silico, contrasting with the prevalent NRPs and PKs BGCs in bacteria.^[^ ^48^ ^]^ Despite these insights, our understanding of archaeal chemical language remains markedly limited compared to our comprehensive knowledge of bacteria and fungi,^[^ ^49^, ^50^ ^]^ particularly regarding their biosynthesis and ecological roles. Although RiPPs exhibit a high diversity and widespread distribution in archaea, class II lanthipeptides have been found to exist exclusively in halophilic archaea, according to the results of our archaeal genomic analysis.^[^ ^29^, ^30^, ^31^ ^]^ This observation suggests archaeal lanthipeptides to be niche‐specific metabolites adapted to hypersaline environments, as evidenced by the amino acid composition bias and phylogenetic analysis of the hallmark enzyme LanMs and their corresponding precursor LanAs in this study. We then employed a combined strategy involving the biosynthetic rule‐guided metabolomic analysis and the first use of heterologous expression of archaeal BGCs in Haloarchaea to unveil the chemical landscape of archaeal lanthipeptides. The heterologous expression approach substantially expands the repertoire of archaeal SMs and encourages further exploration of other unique and silent BGCs from the domain of Archaea, such as diverse and widely distributed YcaO/rSAM‐modified RiPPs and NRPs. However, the inherent challenges, notably incomplete assembly of biosynthetic pathways and enzymatic biases imposed by host‐specific machinery, could significantly impact the resulting metabolites. Consequently, the natural occurrence of these BGC‐derived products remains speculative until their biosynthesis is experimentally confirmed within their native archaeal lineages. Regardless, our identification of diverse archaeal lanthipeptides advances the exploration of their biosynthetic pathways and ecological roles, setting the stage for deciphering other SM‐mediated chemical interactions.

The discovery of a new lanthipeptide subfamily, DADC lanthipeptides, underscores the distinct characteristics of archaeal metabolic pathways and enzymes. Notably, the presence of LL‐MeLan residues in larlans and medlans, which lack the adjacent dehydroamino acid residues typically responsible for controlling this stereochemistry,^[^ ^51^ ^]^ suggests that alternative enzymatic or evolutionary mechanisms may govern LL‐MeLan formation in archaeal lanthipeptides. Furthermore, these lanthipeptides are marked by the unprecedented diamino‐dicarboxylic termini, resulting from the unknown cleavage of amino acids within the thioether rings. This unique biosynthetic feature resembles but differs from the biosynthesis of myxococin B, which was cleaved by a peptidase within the BGC and suspends amino acids along the peptide chains without removing any from the core region.^[^ ^52^ ^]^ Drawing on existing knowledge, we propose that DADC lanthipeptide also undergoes a multiple‐step maturation process, initially forming classical lanthipeptides and then cleaving amino acids within the thioether rings through a generic protease encoded outside the BGCs. This hypothesis is supported by the findings from the in vivo reconstitution of BGC *lar* and the comparative analysis of heterologous expression products with wild‐type natural products derived from BGC *alnβ* (Figure 3). DADC lanthipeptides exhibit bioengineering potential through modifications like methylation and acetylation on archalan β3, indicating the feasibility of engineering at four termini. The precise biosynthetic pathways and enzymes of DADC lanthipeptides remain elusive due to the complexity and challenges encountered in the enzymatic study. Nonetheless, our research sheds light on the complexity and novelty of archaeal SMs, emphasizing the untapped potential of archaea in natural products and enzyme discovery.

The ecological function of archaeal lanthipeptides was evaluated through in vitro antimicrobial testing and in vivo motility assays. Their antagonism against haloarchaea may stem from the prevalence of haloarchaea over normal bacteria in halophilic habitats.^[^ ^43^ ^]^ In addition, archaeal lanthipeptides were found to be involved in activating motility, revealing a previously unknown ecological function of RiPPs. Notably, our microscopic and transcriptomic analyses provide evidence that lanthipeptides enhanced the host's motility by inducing a shift in cell shape toward a rod‐like morphology and upregulating the expression of archaellin gene *flgA1*. These findings underscore the potential role of lanthipeptides in influencing archaeal motility for adapting to the environmental stimuli,^[^ ^44^, ^45^, ^47^ ^]^ despite our limited understanding of regulation mechanisms at the molecular level.

## Conclusion

This study presents the first heterologous expression of SMs in archaea to reveal the chemical landscape of archaeal lanthipeptides with unique biosynthetic features. We have demonstrated a newfound ecological function of archaeal lanthipeptides in regulating motility, with the mechanism involving the transformation of rod‐shaped cell morphology and the upregulation of archaellin expression. Our discovery of structurally novel and biofunctional lanthipeptides from Archaea is anticipated to spur research into the less‐explored field of archaeal chemical biology and chemical ecology.

## Author Contributions

Z.‐M.S. and Y.‐X.L. designed the research and prepared the manuscript. Z.‐M.S., C.C., H.L., J.Z. and Q.Z. performed research. Z.‐M.S. and Y.‐X.L. characterized the structures of compounds. Z.‐M.S. and C.C. performed the LC‐MS analysis. Z.‐M.S., Y.G., X.L., D.Z., and G.W. did the genomic analysis. Y.G. analyzed the transcriptomic data. Z.‐M.S., Y.Q., and H.J. performed the phase‐contrast microscopic analysis. Z.‐M.S., C.C., Y.G., X.L., Y.Q., D.Z., G.W., H.L., Q.Z., J.Z., P.Y. C., H.J., and Y.‐X.L. analyzed the data and discussed the results. W.L. and Y.‐X.L. founded the research. Y.‐X.L. supervised the study. All authors read and approved the final manuscript.

## Conflict of Interests

The authors declare no conflict of interest.

## Supporting information



Supporting Information

Supporting Information

## Data Availability

The data that support the findings of this study are available in the supplementary material of this article.
