# Supplementary material for: Decoding the Chemical Language of Ribosomally Synthesized and Post‐Translationally Modified Peptides from the Untapped Archaea Domain
Source: Angew Chem Int Ed Engl. 2025 Apr 14;64(24):e202501074. doi: 10.1002/anie.202501074 (PMC12144867; doi:10.1002/anie.202501074)
Supplement: Supplementary file 2 — Supporting Information [file ANIE-64-e202501074-s001.zip › Supplementary datasets 1-6/Legends_of_Supplementary_data.docx]

Description of Additional Supplementary data Files：

Supplementary data 1:

The distribution, diversity and novelty of archaeal biosynthetic gene cluster predicted by antiSMASH 7.0.

Supplementary data 2:

The predicted archaeal LanAs and LanMs and their amino acid composition biases compared to bacterial LanAs and LanMs.

Supplementary data 3:

Protein sequence and accession ID of the BGC in this study.

Supplementary data 4:

The motility assay of different recombinant strains.

Supplementary data 5:

The circularity of different recombinant strains.

Supplementary data 6:

Transcriptomic analysis of differential expression genes in different recombinant strains.
